# Supplementary material for: Transcriptional response of the xerotolerant Arthrobacter sp. Helios strain to PEG-induced drought stress
Source: Front Microbiol. 2022 Oct 13;13:1009068. doi: 10.3389/fmicb.2022.1009068 (PMC9608346; doi:10.3389/fmicb.2022.1009068)
Supplement: Supplementary file 1 [file Data_Sheet_1.ZIP › Supplementary figures.docx]

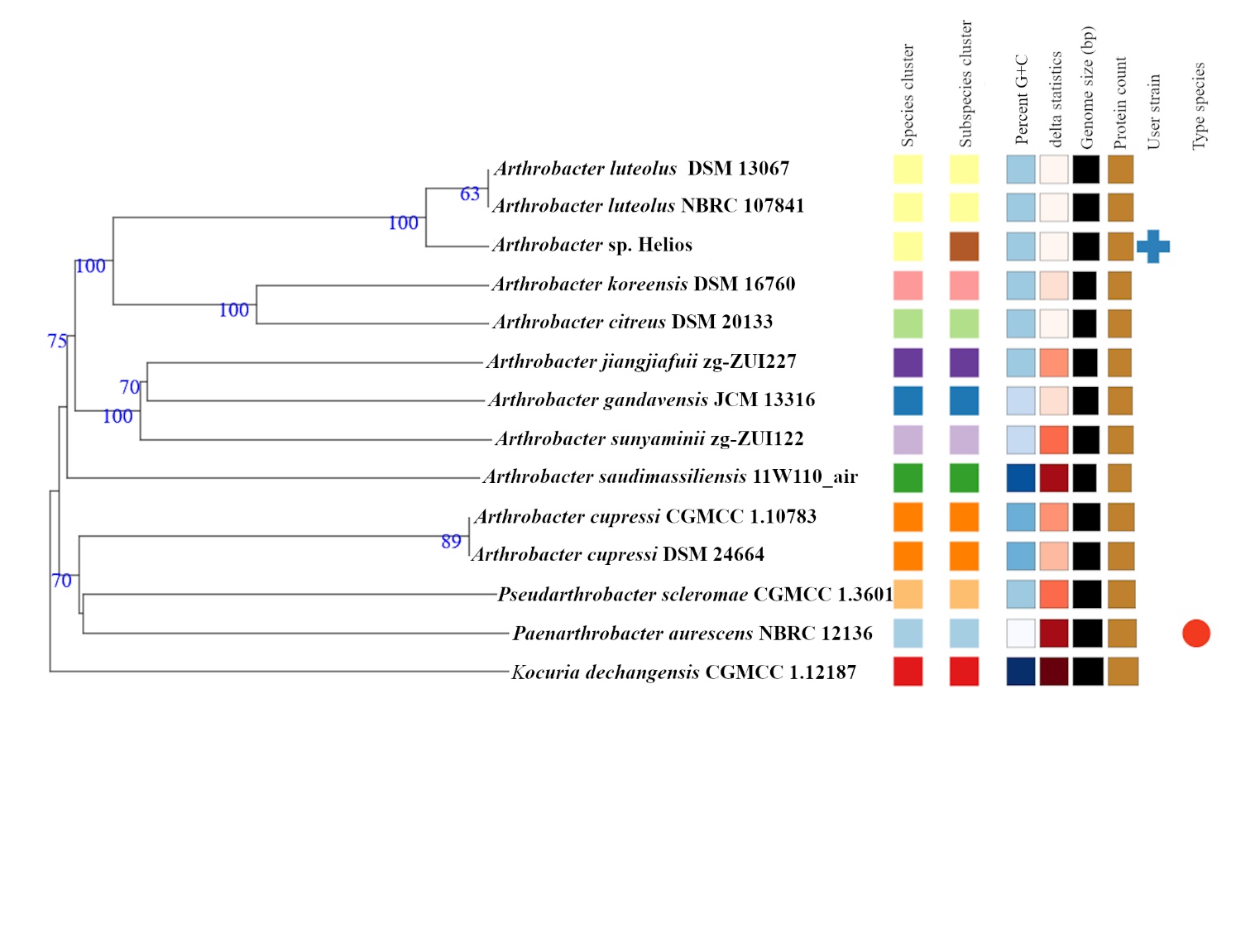


**Supplementary Figure 1.** Phylogenetic whole-genome sequence-based tree performed with the Type Strain Genome Server.


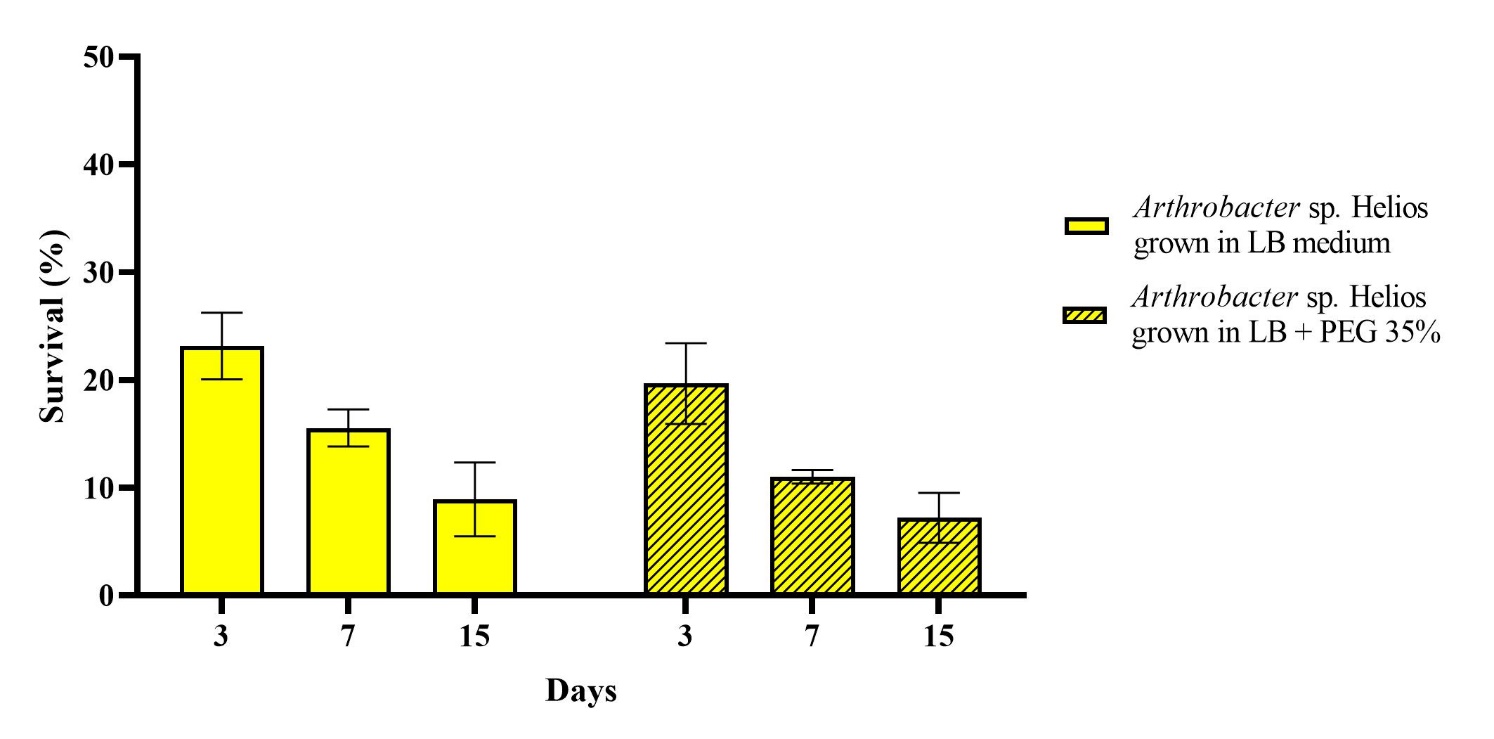


**Supplementary Figure 2.** Desiccation resistance comparison of *Arthrobacter* sp. Helios in stationary phase of growth in LB medium and LB medium with 35% PEG 6000.


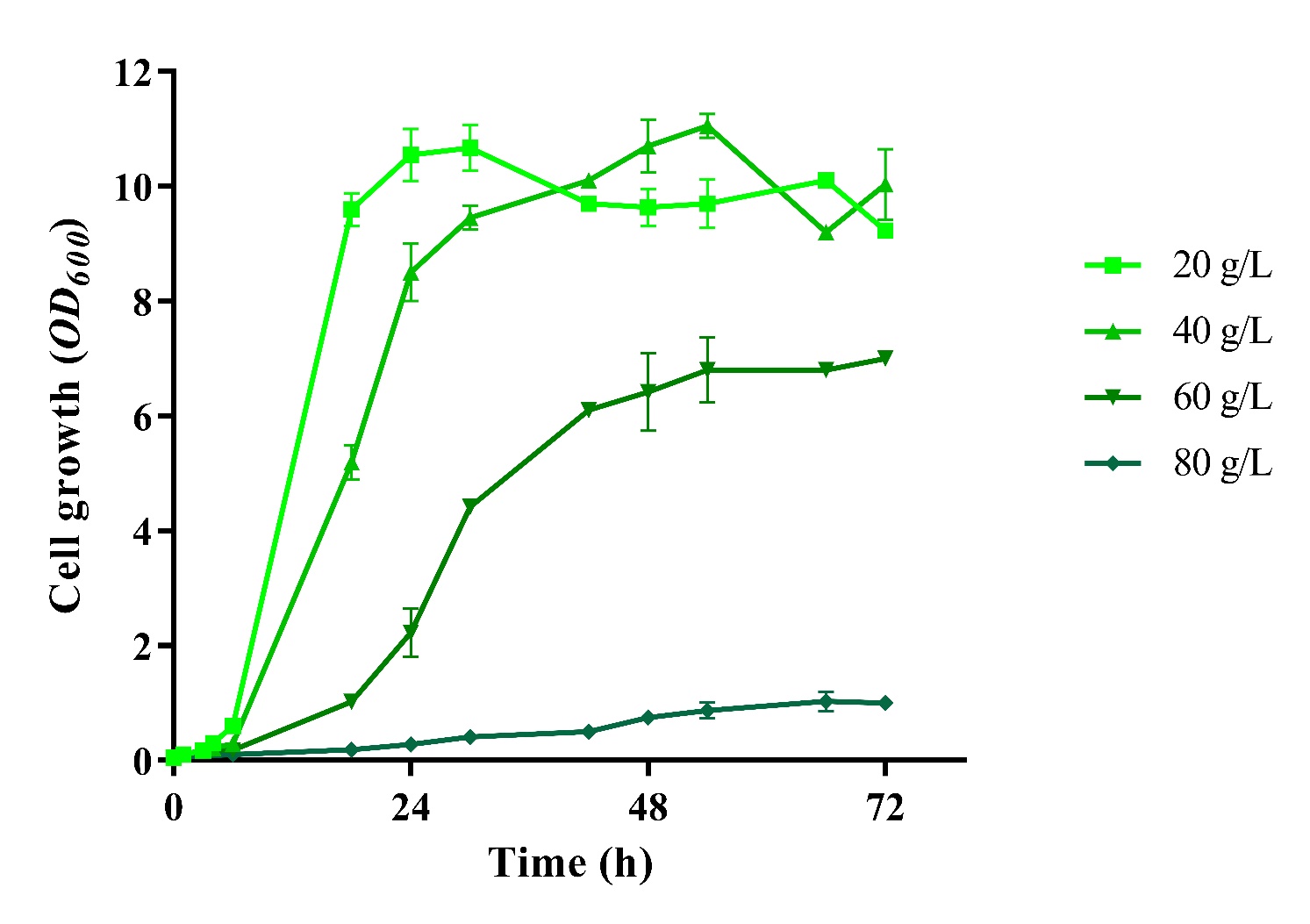


**Supplementary Figure 3.** Growth curve of *Arthrobacter* sp. Helios in LB medium with increasing concentrations of NaCl.


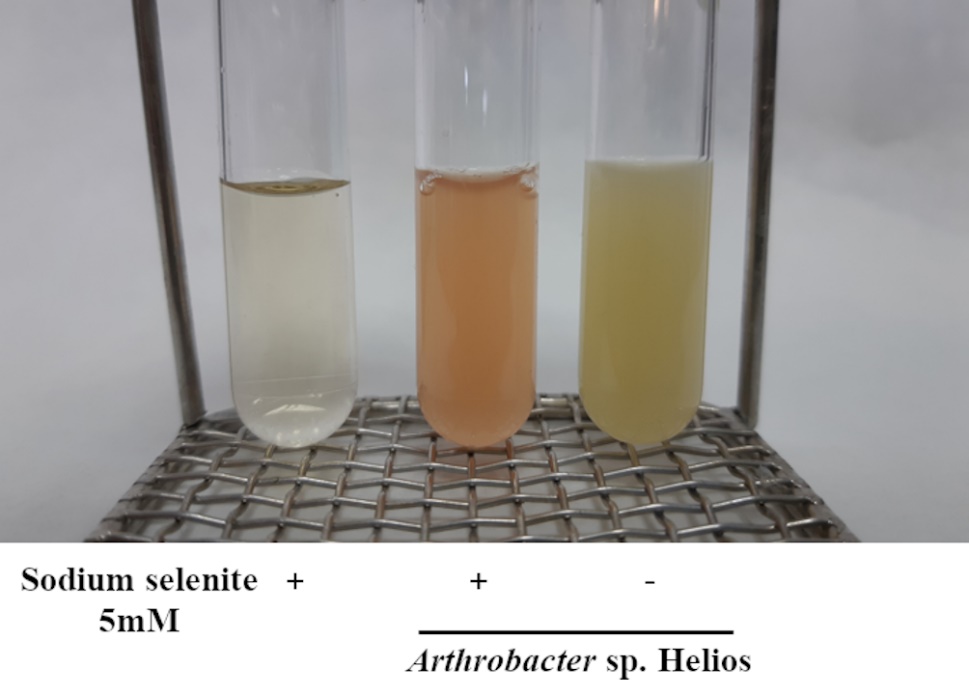


**Supplementary Figure 4.** Sodium selenite reduction by *Arthrobacter* sp. Helios grown in LB medium.

**Supplementary Figure**
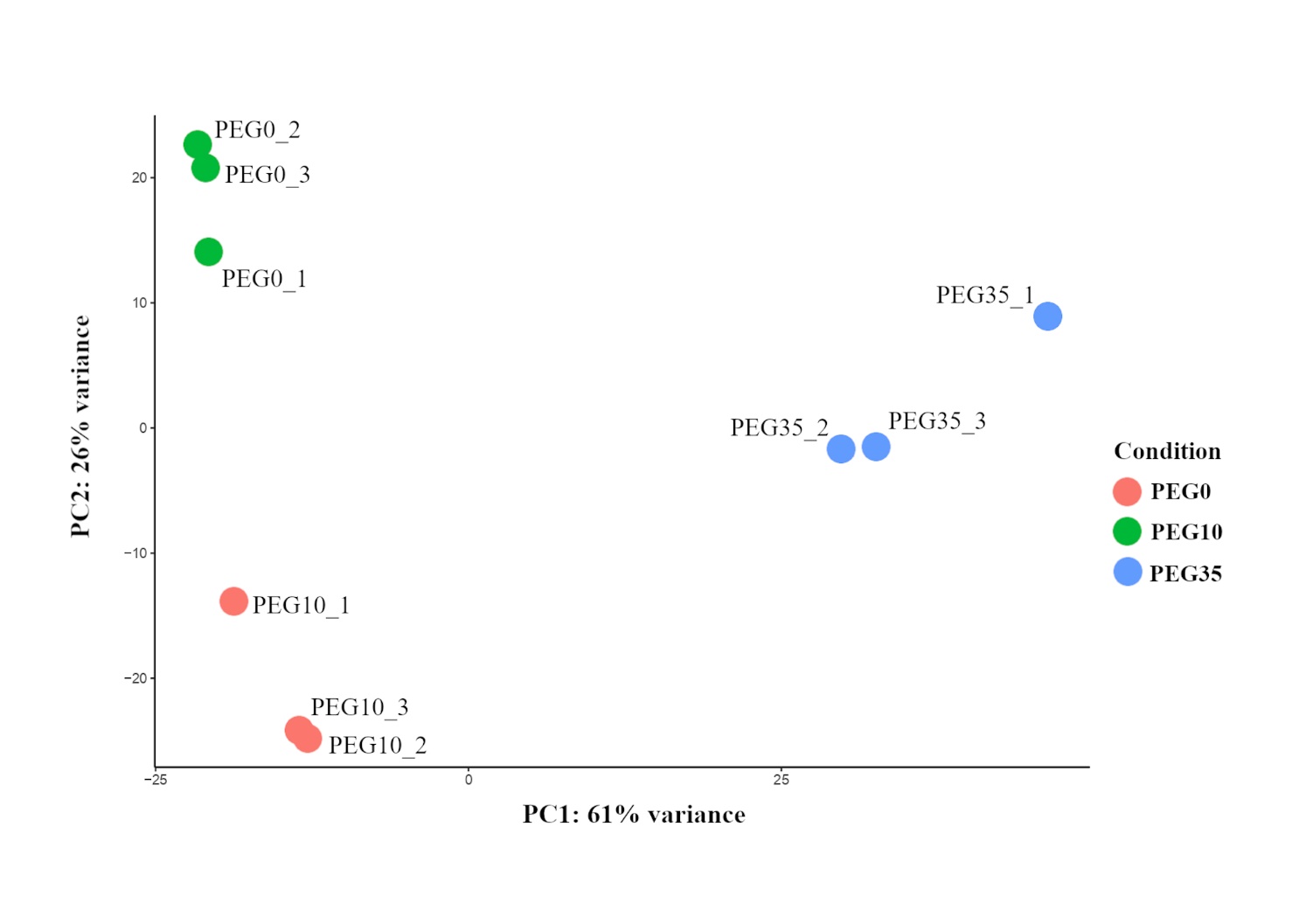
**5.** Principal component analysis. Red, green and blue circles represent triplicates of *Arthrobacter* sp. Helios samples grown in PEG0, PEG10 and PEG35, respectively.
